# Construction of Self-Healing Disulfide-Linked Silicone Elastomers by Thiol Oxidation Coupling Reaction

**DOI:** 10.3390/polym13213729

**Published:** 2021-10-28

**Authors:** Yanhua Huang, Jianpan Yan, Dengxu Wang, Shengyu Feng, Chuanjian Zhou

**Affiliations:** 1 School of Materials Science and Engineering, Shandong University, Jinan 250022, China; hyhsdu@163.com; 2 National Engineering Research Center for Colloidal Materials, Key Laboratory of Special Functional Aggregated Materials, Ministry of Education, Shandong Key Laboratory of Advanced Organosilicon Materials and Technologies, School of Chemistry and Chemical Engineering, Shandong University, Jinan 250100, China; jpanyan@163.com (J.Y.); fsy@sdu.edu.cn (S.F.)

**Keywords:** silicone elastomers, self-healing, thiol oxidation coupling reaction, disulfide bond, adhesives

## Abstract

Developing self-healing silicone elastomers are highly significant because of their promising applications. Herein, we present novel self-healing disulfide-linked silicone elastomers (SEs) based on thiol-terminated sulfur-containing heterochain polysiloxanes (P-SHs) and three thiol-containing crosslinkers, including pentaerythritol tetrakis(β-mercaptopropionate) (PETMP), octa(3-mercaptopropyl)silsesquioxane (POSS-SH), and poly[(mercaptopropyl)methylsiloxane] (PMMS), via the thiol oxidation coupling reactions. The construction of these SEs can rapidly proceed at room temperature. The effects of crosslinker species and amounts on the formability and mechanical properties were investigated. The silicone elastomers can be self-healed by heating at 150 °C for 2 h or under UV radiation for 30 min after cutting them into pieces and the self-healing efficiency is >70%. Moreover, they can be utilized as adhesives for bonding glass sheets, which can hold a 200 g weight. The bonding is reversible and can repeatedly proceed many times, indicating that these materials can promisingly be applied as reversible adhesives. These results indicate that a thiol oxidation coupling reaction is a simple and effective strategy for the construction of self-healing disulfide-linked elastomers. Under this strategy, more disulfide-linked organic elastomers with self-healing properties can be designed and constructed and their applications can be further explored.

## 1. Introduction

Silicone elastomers (SEs) as an important class of organosilicon materials have attracted significant interest due to their intriguing properties, such as high/low temperature resistance, high surface activity, electric resistance, and biocompatibility, and have found extensive applications in aerospace, electronics, coatings, adhesives, medicine, and so forth [1,2,3,4]. Recently, SEs with a self-healing property have gained particular attention because this property not only can lead to the extension of their usable life, recycling, and reduction of waste, but also can meet their high requirements in emerging applications, such as stretchable electronics, wearable strain sensors, and soft robots [5,6,7]. The SEs with a self-healing property are typically realized by pre-embedded a healing agent in the system (called extrinsic self-healing), or incorporating dynamic linkers in the networks (called intrinsic self-healing) [6]. In recent years, most of the efforts have been devoted to develop the intrinsic self-healing SEs because the diversified dynamic interactions, including reversible covalent bonds (such as disulfide [8], boroxine [9], imine [10], a Diels-Alder reaction [11], transesterification [12]), coordination bonds [13], hydrogen bonds [14], π-π stacking interactions [15], and host–guest interactions [16]) can be readily incorporated in the networks and the obtained SEs can be repeatedly healed.

Among various dynamic interactions, disulfide bonds—as a typical reversible bond—have been widely applied to construct self-healing materials as their reversible nature can be easily triggered by various conditions, such as heating [17,18], UV light [19], sunlight [20,21], and alkaline [22]. In addition, by virtue of disulfide bonds, novel self-healing SEs were also achieved [23,24,25]. For example, Zhang et al. reported sulfide-linked SEs using α,ω-dihydroxyl polydimethylsiloxane as the base polymer and commercially available bis(triethoxysilylpropyl) disulfide as the crosslinker [23]. The resultant SEs can be self-healed and recycled multiple times without any catalyst under sunshine. By introducing aromatic disulfides into a siloxane matrix, a stretchable, fast-healing SE exhibiting a tensile stress of 0.5 MPa and a healing efficiency above 95% at room temperature has been constructed [26]. Nevertheless, the disulfide bonds in these SEs were commonly formed before the crosslinking. In other words, disulfide-containing monomers were commonly utilized as the crosslinkers for the SEs. There is still no report on self-healing SEs crosslinked by a disulfide bond, which is directly formed during the crosslinking.

Herein, we introduce a thiol oxidation coupling reaction as the crosslinking strategy to construct disulfide bond crosslinked SEs using thiol-terminated sulfur-containing heterochain polysiloxane as the base polymer and thiol-containing compounds or polymers as the crosslinkers. The mechanical properties of resultant SEs can be tuned by altering the amount and species of crosslinkers. By taking advantages of reversible disulfide bonds in the networks, the SEs can be self-healed under heating or UV irradiation. In addition, their application as adhesives was also explored.

## 2. Materials and Methods

### 2.1. Materials and Characterization

We purchased 1,3-divinyl-1,1′,3,3′-tetramethyldisiloxane (99%), 1,2-ethanedithiol (99%), and 2,2-dimethoxy-2-phenylacetophenone (98%), and pentaerythritol tetrakis(β-mercaptopropionate) (95%) from Energy Chemical (Shanghai, China). Sodium iodide (99%) and 30% H_2_O_2_ aqueous solution were purchased from Tianjin Kemiou Chemical Reagent Co., Ltd. (Tianjin, China) and Laiyang Fine Chemicals (Laiyang, China), respectively. The commercially available reagents were used without further purification. Poly[(mercaptopropyl)methyldimethylsiloxane] (PMMS) was prepared as in our previous report [27]. Octa(3-mercaptopropyl)silsesquioxane (POSS-SH) was synthesized as a previous report and the purity is >99% based on the elemental analysis [28]. Fourier transform infrared (FT-IR) spectra were measured within a 4000 to 400 cm^−1^ region on a Bruker TENSOR-27 infrared spectrophotometer (KBr pellet) (Ettlingen, Germany). ^1^H NMR were measured on a Bruker AVANCE-400 NMR spectrometer (Rheinstetten, Germany) using CDCl_3_ as the solvent and without tetramethylsilane (TMS) as an internal reference. Elemental analyses were conducted using an Elementar vario EL III elemental analyzer (Munich, Germany). Thermogravimetric analysis (TGA) (Mettler Toledo, Columbus, OH, USA) was performed under N_2_ using a TA SDTQ600 at a temperature range of room temperature to 600 °C with a heating rate of 10 °C min^−1^. The differential scanning calorimetry (DSC) (Mettler Toledo, Columbus, OH, USA) was conducted under N_2_ using a Netzsch DSC 204 Phoenix at a temperature range of −100 to 150 °C with a heating rate of 10 °C·min^−1^. The tensile properties of elastomers were tested using the 3340 model Instron universal testing machine at room temperature with a test speed of 100 mm/min.

### 2.2. Synthesis of Thiol-Terminated Sulfur-Heterochain Polysiloxane (P-SH-1 to P-SH-4)

P-SH-1: 1,3-divinyl-1,1′,3,3′-tetramethyldisiloxane (MM^Vi^) (18.64 g, 0.1 mol), 1,2-ethanedithiol (EDT) (11.30 g, 0.12 mol), and 2,2-dimethoxy-2-phenylacetophenone (DMPA) (0.3 g) were added to a 50 mL round-bottom flask. The mixture was stirred for 30 min while being exposed to UV light at a wavelength of 365 nm. The viscosity of the mixture gradually increased. Then cold methanol was added to the mixture to wash the unreacted reagents and DMPA three times. After removing the methanol under vacuum, the product was obtained as a colorless and viscous liquid (yield: 91.5%, 27.40 g).

P-SH-2: the synthesis and post-treatment of P-SH-2 were similar to those of P-SH-1 except that the amount of EDT was 10.36 g (0.11 mol) with a molar ratio of EDT and MM^Vi^ as 1.1:1. The product was obtained as a colorless and viscous liquid (yield: 92.5%, 26.82 g).

P-SH-3: the synthesis and post-treatment of P-SH-3 were similar to those of P-SH-1 except that the amount of EDT was 9.89 g (0.105 mol) with a molar ratio of EDT and MM^Vi^ as 1.05:1. The product was obtained as a colorless and viscous liquid (yield: 92.4%, 26.36 g).

P-SH-4: the synthesis and post-treatment of P-SH-4 were similar to those of P-SH-1 except that the amount of EDT was 9.51 g (0.101 mol) with the molar ratio of EDT and MM^Vi^ as 1.01:1. The product was obtained as a colorless and viscous liquid (yield: 92.4%, 26.36 g).

^1^H NMR (400 MHz, CDCl_3_): *δ* 2.63–2.76 (m, −SCH_2_CH_2_SH), 2.52–2.60 (m, −SiCH_2_CH_2_S–), 1.24–1.29 (m, −SH),0.82–0.90 (m, −SiCH_2_CH_2_S–), 0.01–0.14 (m, −SiCH_3_).

The molecular weights of P-SH-1 to P-SH-4 were 1500, 2900, 5700, and 28,100 g/mol, which were calculated based on the results of ^1^H NMR.

### 2.3. General Synthesis of Disulfide-Linked SEs

In a typical synthesis, P-SH-1 (2 g, 1.34 mmol), pentaerythritol tetrakis(β-mercaptopropionate) (PETMP) (65.4 mg, 0.134 mmol), and tetrahydrofuran (THF) (10 mL) were placed into a weighing bottle and mixed under stirring. Then NaI in aqueous solution (0.05 mL, 0.03 mmol, 0.1 g/mL) and 30% H_2_O_2_ aqueous solution (0.1 mL) were added in the solution and stirred for 50 min at room temperature. The resultant mixture was poured into a Teflon mold and remained at room temperature. The resultant crude elastomer was washed by 5% sodium thiosulfate aqueous solution three times to remove the residual catalyst and oxidant. Finally, the product was dried at 90 °C in a vacuum oven for 24 h and afforded as a transparent elastomer (SE-4). Warning: the 30% H_2_O_2_ aqueous solution should be carefully taken and used because it can potentially cause fires and explosions!

Other SEs were achieved under a similar procedure of SE-4 except for the formulation of the starting materials. Table 1 summarizes the variables that were examined to study the correlation of formability of SEs with the reagents. The factors include the types of P-SHs and crosslinkers, and the amounts of crosslinkers.

### 2.4. Lap Shear Adhesion

The lap shear adhesion referred to a previous report with some modification [18]. Lap joints were made by placing a 6 mm × 6 mm square of elastomer between two overlapping 25 mm × 75 mm glass sheets. The resultant sheets were clamped using two clamps and exposed to UV light for 30 min. During this adhesive process, contact pressure was maintained. After removing the UV irradiation, the adhesive glass sheets were applied to hold different weights.

## 3. Results and Discussion

### 3.1. Synthesis and Characterization

**Scheme 1:**
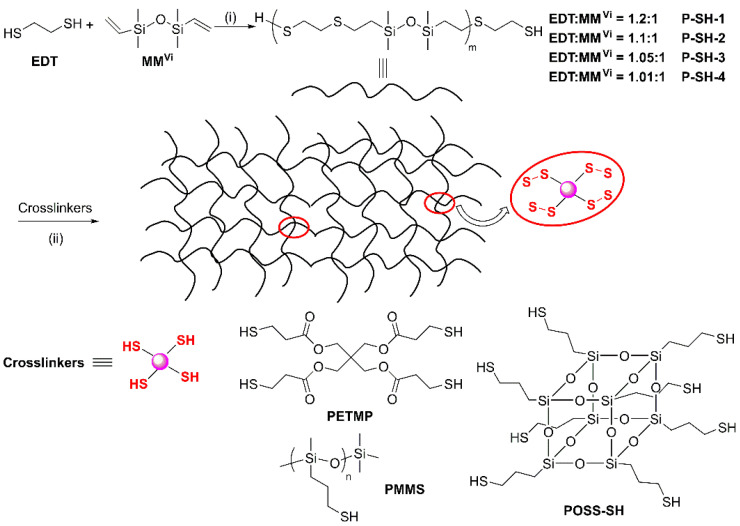
Synthesis of disulfide-linked SEs. (i) DMPA, UV light, room temperature, 30 min; (ii) NaI, 30% H_2_O_2_ aqueous solution, THF, room temperature.

As shown in Scheme 1, the thiol-terminated sulfur-containing heterochain polysiloxane (P-SHs) were firstly synthesized by the thiol-ene reaction of 1,3-divinyl-1,1′,3,3′-tetramethyldisiloxane (MM^Vi^) and 1,2-ethanedithiol (EDT) with DMPA as the initiator under UV-light. To control the molecular weights of P-SHs, the molar ratios of EDT and MM^Vi^ were altered from 1.2:1 to 1.01:1. Then the sulfide-linked SEs were synthesized by the thiol oxidation coupling reactions, while using P-SHs as the base polymers and three thiol-containing compounds or polymers, including PETMP, POSS-SH, and PMMS, as the crosslinkers. It was found that the crosslinking process can rapidly occur at room temperature. After removing the unreacted reagents and catalyst, the final products were afforded and the formability of these elastomers depends on the formulations (Table 1, see the following discussion).

**Figure 1:**
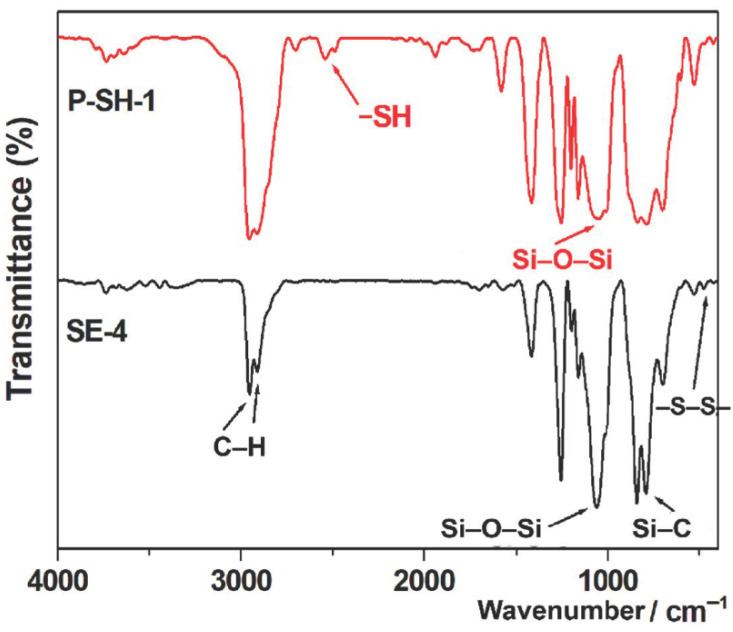
FT-IR of P-SH-1 and SE-4.

The structure of SEs was determined by FT-IR spectroscopy (with SE-4 as an example). As shown in Figure 1, the peaks at 2963, 2913, 1064, 790, and 700 cm^−1^ in SE-4 can be attributed to –CH, –Si–O–Si–, –Si–C– and –C–S– stretching vibrations. Compared to P-SH-1, the peak at ca. 2540 cm^−1^ assigned to –SH groups disappeared in the silicone elastomer, while a new and weak peak at ca. 472 cm^−1^, attributable to –S–S– stretching vibration, was observed. This finding indicates that the –SH groups were oxidized to –S–S– bonds, thus resulting in the formation of the crosslinking network. These results were consistent with our recent report [27].

### 3.2. Effects of Variables on the Formability and Mechanical Properties of SEs

As mentioned above, the SEs were prepared by the thiol oxidation coupling reactions. To form a silicone elastomer with good quality, the crosslinking degree should be appropriate. If the degree is too low, the elastomers cannot be formed and the products appear as a viscous liquid. If the degree is too high, the products may be afforded a hard plastic with cracks. Thus, the effects of the amounts and species of crosslinkers on the formability and the mechanical properties of the resultant SEs were investigated.

Three crosslinkers, including PETMP, POSS-SH, and PMMS, were utilized to investigate the effects. As expected, the SEs cannot be afforded in the absence of crosslinker or with low amount of crosslinkers (e.g., SE-1, SE-2, SE-11, SE-15, and SE-19 in Table 1). For example, the products (SE-1 and SE-2) were tacky when the amounts of PETMP were zero or 0.016 g and the other parameter included 2 g of P-SH-1, 8 mL of THF, 0.1 mL of 30% H_2_O_2_ in aqueous solution, and 0.05 mL of NaI in aqueous solutions (0.1 g/mL). However, when the amount of PETMP is 0.1 g, the elastomer can be afforded, but with many cracks (SE-5). Similar results were also found when using POSS-SH as the crosslinker (SE-6 to SE-9). However, when using PMMS, the elastomers cannot be afforded even if the amount of PMMS is 0.1 g (SE-10). This finding may be due to its high molecular weight. Unlike the small molecular crosslinkers PETMP and POSS-SH, PMMS with high molecular weight cannot effectively link the base polymer P-SHs, but might link themselves, thus leading to unsuccessful formation of the crosslinking network. In addition, when selecting P-SH-2 or P-SH-3 as the base polymers, the amount of crosslinkers on the formability of SEs were similar (SE-11 to SE-14 for P-SH-2, SE-15 to SE-21 for P-SH-3). Thus PETMP and POSS-SH were selected as the crosslinkers to investigate the effects. It is worthy to note that when using P-SH-4 as the base polymer, the SE cannot be formed even selecting the mixture of PETMP and POSS-SH as the crosslinker, and the product was afforded as a tacky liquid (SE-22). This finding can be explained by the low reactivity of –SH of P-SH-4 due to its high molecular weight and the thiol oxidation coupling reaction might mainly occur among the crosslinkers, not between the crosslinkers and P-SH-4.

**Figure 2:**
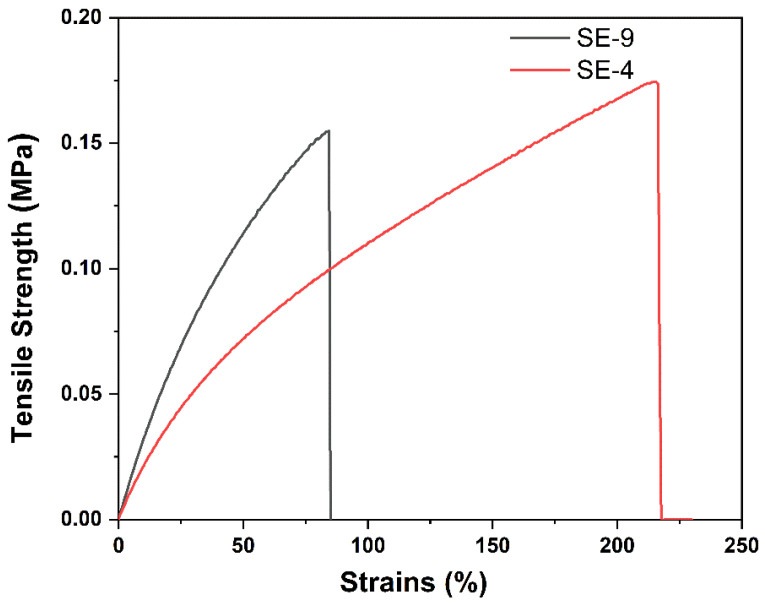
Tensile curves of SE-4 and SE-9.

The effect of crosslinker species on the mechanical properties was studied. SE-4 and SE-9 were selected because the contents of –SH groups based on PETMP and POSS-SH are nearly same. The results show that SE-4 with PETMP has a higher tensile strength of 0.17 MPa and the elongation at break (218%) than those (0.15 MPa and 85%) of SE-9 with POSS-SH (Figure 2). This finding is apparently due to the different structure of crosslinkers. POSS-SH is a rigid and cage molecule and has higher rigidity than PETMP. Thus, the formed crosslinking network is difficult to rotate. Moreover, POSS-SH can provide more crosslinking sites than PETMP. As a result, when using POSS-SH as a crosslinker, a rigid and concentrated crosslinking was formed in SE-4 and SE-4 exhibits a better mechanical property than SE-9.

**Figure 3:**
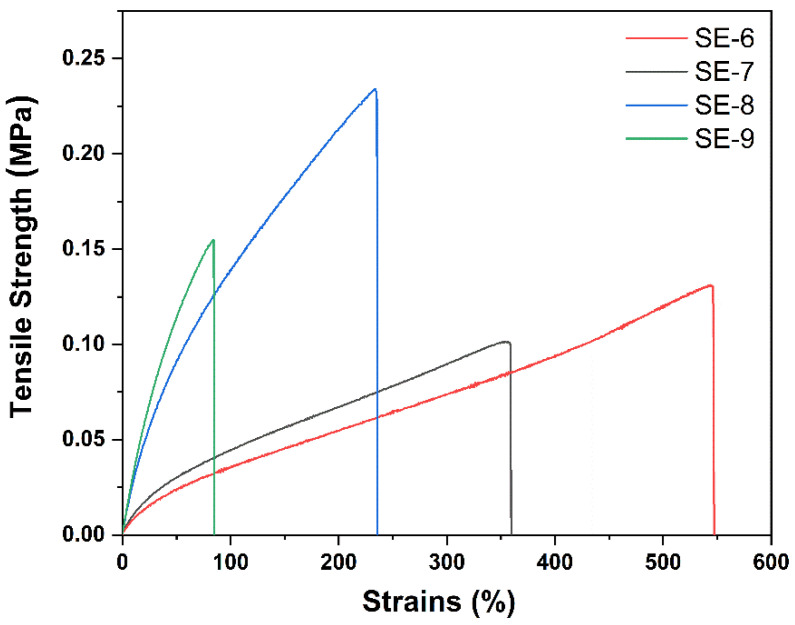
The effect of the cross-linker content on stress of the elastomer.

The effect of the amount of crosslinkers on the mechanical properties was further studied and the elastomers with POSS-SH as the crosslinker were selected as examples (Figure 3). With an increment of the amount of POSS-SH, the elongation at break gradually decreased from 545% (SE-6) to 85% (SE-9), while the trend change of the tensile strength was not regular and SE-8 with the moderate amount of crosslinker exhibited the highest tensile strength of 0.23 MPa. This finding can be explained by the tuned crosslinking density induced by the varied amount of crosslinker. It is known that when increasing the amount of crosslinker, the crosslinking sites would increase, leading to enhanced tensile strength and elongation at break. However, if the amount is too high, the excessive crosslinking will lead to cracks in the elastomer and make the tensile strength decrease.

### 3.3. Thermal Stability

**Figure 4:**
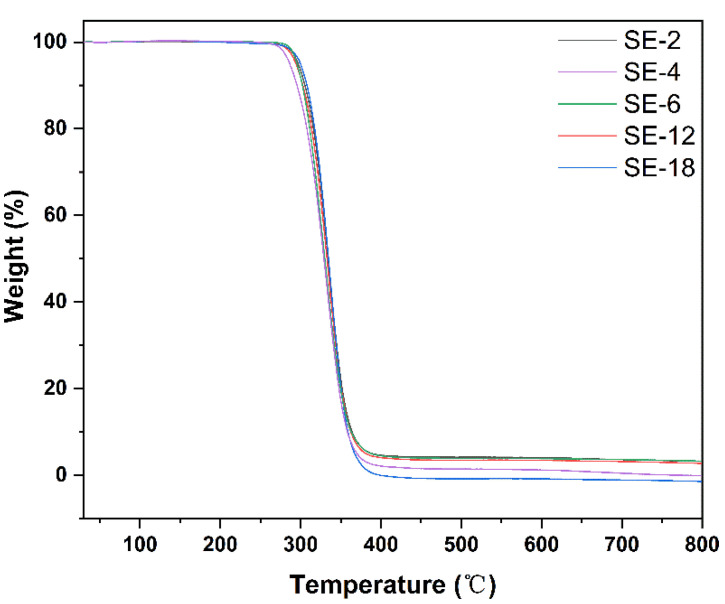
TGA curves of SE-2, SE-4, SE-6, SE-12 and SF-18 at a heating rate of 10 °C min^−1^ in nitrogen.

The thermal stability of these elastomers was evaluated by thermogravimetric analysis (TGA) and differential scanning calorimetry (DSC) because it plays an important role in their applications. The TGA experiments were conducted at a heating rate of 10 K/min under N_2_. Figure 4 shows the TGA curves of SE-2, SE-4, SE-6, SE-12, and SE-18 as examples. It was found that these elastomers exhibit high thermal stability with the T_d,5%_ (5% weight loss temperature) at ca. 300 °C. After that, the elastomers quickly decompose and fully decompose at ca. 400 °C. This finding may be due to the decomposition of disulfide bonds, which induced the decomposition of sulfur-containing heterochain polysiloxane and the formation of some cyclic monomers.

**Figure 5:**
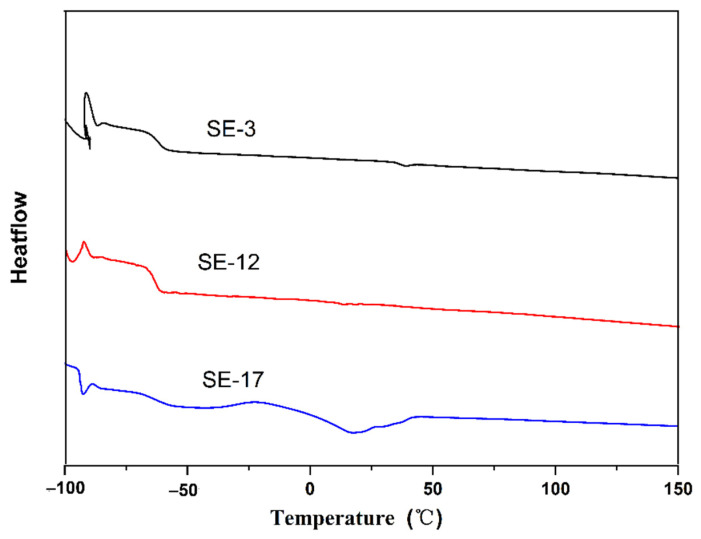
The DSC curves of SE-3, SE-12, and SE-17.

Figure 5 shows the DSC curves of SE-3, SE-12, and SE-17 as examples. The glass transition temperatures of these elastomers are at ca. −60 °C, indicating their low temperature resistance and application in low temperature circumstances. In addition, SE-3 and SE-17 exhibit a crystalline peak at 39 and 20 °C, respectively. The finding is apparently due to the heterochain polysiloxane, which is different from the conventional pure polysiloxane chain [4].

### 3.4. Self-Healing Properties of SEs

**Figure 6:**
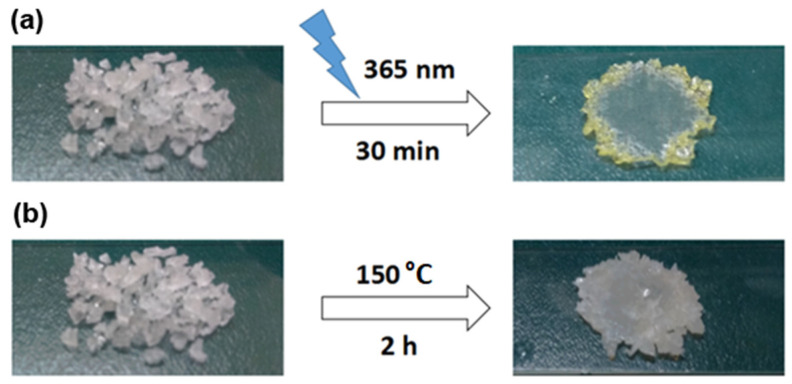
Self-repairing experiments of SE-3 under UV light (365 nm) for 30 min (**a**) or heating at 150 °C for 2 h (**b**).

Due to the presence of reversible disulfide bonds in the networks, these SEs can be expected to possess self-healing properties. Thus, the self-healing property was evaluated using SE-3 as an example. UV light and heating were applied as the healing conditions. Firstly, the elastomer was cut into pieces with a knife. The resultant pieces were placed between two glass sheets and the sheets were clamped. After exposing the pieces to UV light (the wavelength was 365 nm) for 30 min or heating them at 150 °C for 2 h, the elastomers re-formed (Figure 6). These results indicate that the elastomer can be healed.

**Figure 7:**
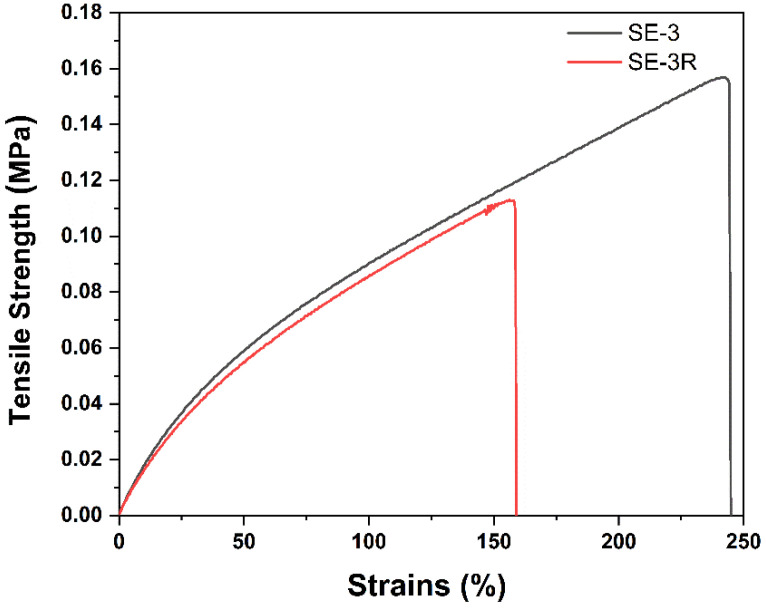
Stress-strain curves before and after self-healing of SE-3. SE-3R represents the elastomer after repair.

The healing efficiency was further evaluated using SE-3 as an example. It was found that if there was no pressure force during the self-healing process under UV light, the elastomer could not be effectively repaired. However, under heating conditions at 150 °C for 2 h, the elastomer can be effectively healed. As shown in Figure 7, the tensile strength is 0.11 MPa, which is lower than the original value of 0.15 MPa. This result means the self-healing efficiency is >70%, thus indicating that the present elastomer can be effectively healed after the destruction.

### 3.5. Application as Adhesives

**Figure 8:**
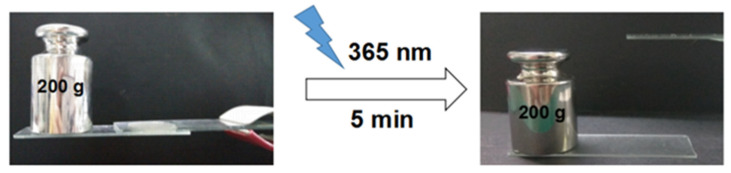
The bonding performance test.

These elastomers were applied as adhesives and SE-4 was selected as an example. The experiment was conducted by placing a 6 mm × 6 mm elastomer between two overlapping 25 mm × 75 mm glass sheets. The resultant sheets were clamped using two clamps and exposed to UV light for 30 min while maintaining the contact pressure. After removing the UV irradiation, the adhesive glass sheets can hold a maximum 200 g of weight, while the glass sheets will break under higher weight. Then the glass sheets were re-exposed to UV light, and the weight dropped because of the decreased adhesive strength (Figure 8). This finding is apparently due to the reversible crosslinking network in the presence of dynamic disulfide bonds. It is delightful that the adhesive and detached process can be repeated many times. These results suggest that these elastomers can be promisingly applied as reversible adhesives.

## 4. Conclusions

Novel self-healing disulfide-linked silicone elastomers have been successfully created by introducing a thiol oxidation coupling reaction as a crosslinking strategy when using thiol-terminated sulfur-containing heterochain polysiloxanes (P-SHs) as the base polymers and three thiol-containing compounds or polymers, including PETMP, POSS-SH, and PMMS, as the crosslinkers. The crosslinking process is mild and rapid. The effects of the crosslinker species and amounts on the formability and mechanical properties were investigated. The SEs can be readily repaired by heating at 150 °C for 2 h or exposure to UV radiation for 30 min with a healing efficiency of > 70%, indicating their self-healing property. For application, they can be used as reversible adhesives for bonding glass sheets, which can hold 200 g of weight. To the best of our knowledge, this work represents the first example of a thiol oxidation coupling reaction as a crosslinking strategy for the construction of self-healing silicone elastomers. This simple and effective strategy can certainly be expanded to construct other self-healing elastomers based on thiol-containing base polymers and crosslinkers and explore the applications of these novel materials.

## Figures and Tables

**Table 1:** Experimental data on the preparation of silicone elastomers under different conditions.

Entry	P-SHs *^a^*	THF/mL	H_2_O_2/_mL *^b^*	NaI mL *^c^*	Crosslinkers (g)	Formability
SE-1	P-SH-1	8	0.1	0.05	PETMP(0)	× (tacky)
SE-2	P-SH-1	8	0.1	0.05	PETMP(0.016)	× (tacky)
SE-3	P-SH-1	8	0.1	0.05	PETMP(0.036)	√
SE-4	P-SH-1	8	0.1	0.05	PETMP(0.065)	√
SE-5	P-SH-1	8	0.1	0.05	PETMP(0.1)	√ (cracks inside)
SE-6	P-SH-1	8	0.1	0.05	POSS-SH(0.01)	√
SE-7	P-SH-1	8	0.1	0.05	POSS-SH(0.02)	√
SE-8	P-SH-1	8	0.1	0.05	POSS-SH(0.04)	√
SE-9	P-SH-1	8	0.1	0.05	POSS-SH(0.06)	√
SE-10	P-SH-1	8	0.1	0.05	PMMS(0.10)	×
SE-11	P-SH-2	8	0.1	0.05	PETMP(0.0168)	× (tacky, bubbles inside)
SE-12	P-SH-2	8	0.1	0.05	PETMP(0.036)	√
SE-13	P-SH-2	8	0.1	0.05	PETMP(0.068)	√
SE-14	P-SH-2	8	0.1	0.05	POSS-SH(0.06)	√
SE-15	P-SH-3	8	0.1	0.05	PETMP(0.0168)	× (tacky, bubbles inside)
SE-16	P-SH-3	8	0.1	0.05	PETMP(0.036)	√ (bubbles inside)
SE-17	P-SH-3	8	0.05	0.05	PETMP(0.036)	√
SE-18	P-SH-3	8	0.05	0.05	PETMP(0.068)	√ (bubbles inside)
SE-19	P-SH-3	8	0.05	0.05	POSS-SH(0.02)	× (tacky, bubbles inside)
SE-20	P-SH-3	8	0.05	0.05	POSS-SH(0.04)	√
SE-21	P-SH-3	8	0.05	0.05	POSS-SH(0.06)	√ (cracks inside)
SE-22	P-SH-4	8	0.1	0.05	PETMP/POSS-SH	× (tacky)

*^a^* All the amounts of P-SHs used in the formulations are 2 g; *^b^* the concentration of H_2_O_2_ in aqueous solution is 30%; *^c^* the concentration of NaI in aqueous solution is 0.1 g/mL. “×” and “√” mean that the elastomers cannot be formed and be formable.

## Data Availability

Not applicable.
